# Identification of nontuberculous mycobacteria species by multiplex real-time PCR with high-resolution melting

**DOI:** 10.1590/0037-8682-0211-2020

**Published:** 2020-11-06

**Authors:** Aline dos Santos Peixoto, Lílian Maria Lapa Montenegro, Andrea Santos Lima, Fábio Lopes Melo, Walter Lins Barbosa, Maria Madileuza Carneiro Neves, Jesus Pais Ramos, Haiana Charifker Schindler, Zulma Maria Medeiros

**Affiliations:** 1 Fundação Oswaldo Cruz, Instituto Aggeu Magalhães, Departamento de Imunologia, Recife, PE, Brasil.; 2 Universidade de Pernambuco, Pós-Graduação em Ciências da Saúde, Recife, PE, Brasil.; 3 Fundação Oswaldo Cruz, Instituto Aggeu Magalhães, Departamento de Parasitologia, Recife, PE, Brasil.; 4 Laboratório Central de Saúde Pública Dr. Milton Bezerra Sobral, Recife, PE, Brasil.; 5 Centro de Referência Professor Hélio Fraga, Escola Nacional de Saúde Pública, Laboratório Nacional de Referência para Tuberculose, Rio de Janeiro, RJ, Brasil.

**Keywords:** Identification, Non-tuberculous mycobacteria, PCR multiplex real-time, Diagnosis, Brazil

## Abstract

**INTRODUCTION:**

Nontuberculous mycobacteria (NTM) species, as human pathogens, are increasing in the world, as is the difficulty of accurately identifying them. Differential diagnosis, especially between the *M. tuberculosis* complex and NTM species, and the characterization of NTM species is important. This study aimed to evaluate the performance of a molecular system based on multiplex real-time PCR with high-resolution melting (HRM) for the identification and differentiation of NTM species of clinical importance of an endemic area for tuberculosis in northeastern Brazil.

**METHODS:**

The technical protocol of the molecular system was based on multiplex real-time PCR-HRM, and evaluated the sensitivity and specificity of the detection of NTM species in mycobacterial clinical isolates from the studied region. The gold standard method was specific gene sequencing.

**RESULTS:**

The sensitivity and specificity of multiplex real-time PCR-HRM modified for differentiation between NTM and *M. tuberculosis* were 90% and 100%, respectively. The PCR-HRM sensitivities for the characterization of NTM species (*M. kansasii, M. abscesses, M. avium, and M. fortuitum*) were 94.59%, 80%, 57.14%, and 54%, respectively.

**CONCLUSIONS:**

The multiplex real-time PCR-HRM modified assay has the potential to rapidly and efficiently identify nontuberculous mycobacteria of clinical importance, which is crucial for immediate implementation of the appropriate therapy and thus avoiding complications and sequelae in patients.

## INTRODUCTION

Nontuberculous mycobacteria (NTM) consist of species of the genus *Mycobacterium*, but with distinct characteristics from *Mycobacterium tuberculosis* ^1^. Tuberculosis (TB) remains a serious public health problem; however, infections caused by nontuberculous mycobacteria have been increasingly recognized as pathologies in humans^2^ ^,^ ^3^. NTM are opportunistic organisms, with more than 199 species and 14 subspecies described, which can be found in soil or water. They are opportunistic pathogens in humans, causing a wide variety of skin and soft tissue infections, lymphadenitis, and lung disease in immunocompromised and immunocompetent individuals^4^ ^,^ ^5^ ^,^ ^6^.

Data from recent studies have demonstrated an increasing prevalence of NTM infections around the world, especially associated with immunosuppression and post-surgical infections^3^ ^,^ ^7^. The increase in NTM infections is worrisome, mainly due to the diversity of species found and the symptom similarity with infections caused by complex *M. tuberculosis* ^8^. The epidemiology of NTM infection differs among countries, and the data vary widely. Therefore, it is important to characterize the distribution of NTM infection by demographics to optimize disease control.

In Brazil, a country with high endemicity for TB (20^th^ position according to WHO classification), the prevalence and incidence of NTM infections are not well known since the disease does not require compulsory notification for the health system. The isolation of NTM species in Brazil differs according to the region; however, it is possible to observe a predominance of *Mycobacterium avium* complex (MAC), such as *M. avium*, and *M. intracellulare*, *M. kansasii*, and *M. fortuitum*, causing active NTM disease^1^ ^,^ ^9^ ^,^ ^10^.

The correct differentiation between *M. tuberculosis* complex and NTM infections is crucial for the immediate implementation of the appropriate therapy. Although classical methods for the identification of mycobacterial species are still used, they are limited. The gold standard method for the identification of NTM species is DNA sequencing^11^, owing to its highly discriminatory nature. However, equipment and running costs are high and are not available in most laboratories in Brazil. The *hsp65, rpoB*, and *16S rRNA* genes are more commonly used for sequencing assays^12^.

Laboratory methods for improving the detection of NTM species are needed because it is important to identify the species that cause NTM infection to start specific treatment and thus avoid complications and sequelae in patients. Thus, we adapted and evaluated the performance of a multiplex real-time PCR with high resolution melting (HRM) assay^13^ for the accurate and rapid identification of NTM species isolated from cultures of clinical importance from a northeastern region of Brazil with a high prevalence of tuberculosis. The study was developed to evaluate a specific molecular identification method to be used as clinical and laboratory support.

## METHODS

This study was descriptive and analysis was carried out to evaluate the performance of the PCR-HRM multiplex real-time technique in relation to DNA sequencing (gold standard) in strains of *Mycobacterium* sp. obtained from culture in Löwenstein-Jensen medium, initially found by phenotypic and biochemical tests in the identification and characterization of NTM species for diagnostic definition.

### *Mycobacterium* species reference strains and cultures of suspected mycobacteriosis

Reference strains of eight *Mycobacterium* species were used, provided by the American Type Culture Collection (ATCC) and the National Institute for Quality Control in Health-INCQS/FIOCRUZ: *Mycobacterium* species reference (*M. tuberculosis* ATCC 25618 (INCQS 00136); *Mycobacterium avium* ATCC 25291(INCQS 00273); *Mycobacterium fortuitum* ATCC 6841 (INCQS 00142); *Mycobacterium intracelullare* ATCC 13950; *Mycobacterium kansasii* ATCC 12478 (INCQS 00134); *Mycobacterium abscessus* ATCC 19977 (INCQS 00631); *Mycobacterium chelone* ATCC 35752; and *Mycobacterium smegmatis* ATCC 607 (INCQS 00061).

Seventy-nine isolates from cultures of mycobacteriosis were provided by laboratory diagnosis in Pernambuco, Brazil (LACEN-PE-Brazil) between January 2012 and June 2014 and were defined based on the American Thoracic Society (ATS)^14^. These isolates were kept in Löwenstein-Jensen medium and incubated at 37 °C for eight weeks^15^ ^,^ ^16^.

### DNA extraction

After growth in Löwenstein-Jensen medium, *Mycobacterium* colonies were suspended in 200 μL of ultrapure water, boiled for 20 min at 99 °C, then frozen at -20 °C for another 20 min. They were centrifuged for pellet formation and stored in a freezer at -80 °C, according to the National Manual of Tuberculosis Vigilance and other Mycobacteria^16^. The pellet was used in the real-time PCR assay.

### Multiplex real-time PCR with high-resolution melting (HRM) method

Genomic DNA extracted from the *Mycobacterium* reference strains and mycobacterial culture isolates was identified using a multiplex real-time PCR-HRM assay, previously developed^13^, with modifications performed. The modifications involved the use of a combination of primers in the different steps of the amplification reactions. They were performed to initially differentiate between *Mycobacterium tuberculosis* and nontuberculous mycobacteria, because this region has a high endemicity for TB. In addition, the following steps were performed to identify NTM species based on the frequency and distribution of those frequently isolated by the reference laboratory for mycobacterioses from Pernambuco, Brazil.

Real-time multiplex PCR-HRM was performed in three steps. Step 1: differentiation between *M. tuberculosis* and NTM, in which the primers used in this reaction were *M. tuberculosis*, NTM, and internal control. The bacterial *16S rRNA* gene was chosen as a target gene for the internal control^2^ ^,^ ^15^. Step 2: identification of NTM species detected in Step 1. In Mix II, primers were used to differentiate the species *M. avium*, *M. intracellulare, M. kansasii,* and *M. abscessus.* Step 3: identification of NTMs that were not identified in Step 2. In Mix III, the primers were used only for *M. fortuitum, M. chelonae,* and M. smegmatis (Table 1).

**TABLE 1:** Targets and primers used in multiplex real-time PCR-HRM.

	PRIMER	Target	5′→3′	Reference	*Tm*
MIX I	Internal control	*16S rRNA*	ATGGCTGTCGTCAGCTCGTG	Cha, 2014; Amann et al.,1995	80.08 ±0.36
			GCTCGTTGCGGGACTTAACC		
	MTC	IS6110	CGAACTCAAGGAGCACATCAG	Kim, 2012	84.47 ±0.41
			CAGGGTTAGCCACACTTTGC		
	NTM	*16S rRNA*	ATGTYTTSTGGKGSAAAGCTTT	Kim, 2012	88.14 ±0.51
			GTAGGAGTCTGGGCCGTA		
MIX II	*M.avium*	*16S rRNA*	GGGTCTAATACCGGATAGGACCT	Kim, 2012	73.55 ±0.34
			CGCAAAAGCTTTCCACCAGA		
	*M.intracellulare*	*16S rRNA*	GGTCTAATACCGGATAGGACCTTTAG	Kim, 2012	75.38 ±0.31
			GCAAAAGCTTTCCACCAAA		
	*M.kansasii*	*16S rRNA*	CGGAAAGGTCTCTTCGGAGAC	Kim, 2012	81.42 ±0.20
			TTTCCCAGGCTTATCCTGGT		
	*M.abscessus*	ITS	ATGAACTAGGGAACATAAAGTATGCA	Kim, 2012	72.72 ±0.74
			AGGATTTACAAAACATATTCACCAAGT		
MIX III	*M. fortuitum*	ITS	CCCGAGCCGTGAGGAAC	Kim, 2012	81.65 ±0.26
			CAATAGTGTGTCTGGCAGTCAAAA		
	*M.chelonae*	ITS	TGTCCACCCCGTGGATA	Kim, 2012	79.40 ±0.25
			GTGCCAGCGTTTCAATTCTA		
	*M.smegmatis*	ITS	GAGCTGGAGCGCTGTAGTG	Kim, 2012	84.28 ±0.14
			GAAACAGCGTTTCCCACAC		

**MTC:** Mycobacterium tuberculosis complex*;* **NTM:** Nontuberculous mycobacteria.

Real-time multiplex PCR-HRM reactions were performed on a Rotor Gene 600S (Corbett Research, Cambridge, United Kingdom)) thermal cycler and contained 10 mmol/L primer mix, 2 × GRH PCR master mix (Qiagen Inc., Germantown, MD, USA), which included EvaGreen fluorescent dye, and 1.5 μL genomic DNA in a final volume of 10 μL. All reactions were performed in duplicate. Positive and negative controls and no-template controls were included in each run. The analytical sensitivity of the assay was determined using a 10-fold dilution (10 ng, 1 ng, 100 pg, 10 pg, 1 pg, 100 fg, 10 fg (equivalent to one bacillus) and 1 fg) standard curve of DNA isolated from *M. tuberculosis* H37Rv. The detection limit of the real-time PCR assay was 1 fg, equivalent to less than one bacillus (data not shown).

### Standardization of melting temperatures (*Tm*) 

**FIGURE 1:**
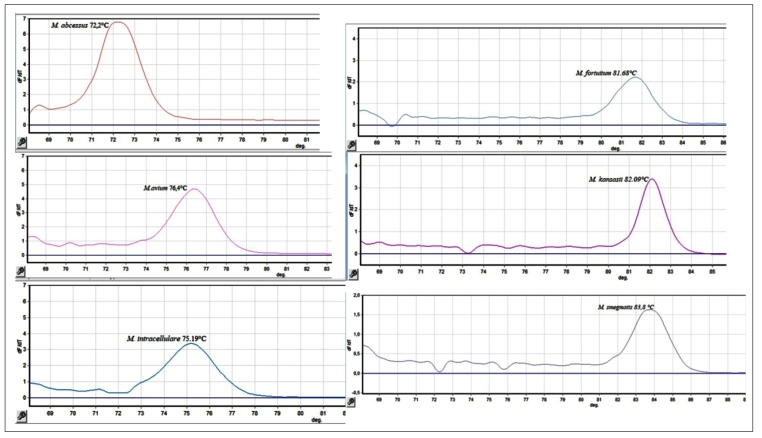
Standardization of melting temperatures in multiplex real-time PCR-HRM nontuberculous mycobacteria.

The assay was initially evaluated using 8 reference strains and was performed using the standardized melting temperatures for multiplex real-time PCR-HRM reactions^13^. All experiments were performed in duplicate (Figure 1).

### Sequencing

The *hsp65, rpoB,* and *16S rRNA* genes were sequenced^17^ ^,^ ^18^ ^,^ ^19^. The amplified products were purified with the GFX PCR Kit for DNA and Gel Band Purification (GE Healthcare, Buckinghamshire, UK) according to the manufacturer's recommendations. Sequencing of the 79 clinical strains was performed on an ABI3130 (Life Technologies, California, United States) using the National Reference Laboratory for Tuberculosis of the Reference Center Professor Hélio Fraga/ENSP/FIOCRUZ. All sequences obtained were compared on the basis of similarity to those in the GenBank database using BLAST for species identification (http://blast.ncbi.nlm.nih.gov/Blast.cgi).

### Data analysis

Specific gene sequencing was considered the gold standard for calculating real-time multiplex PCR-HRM performance. Statistical analysis was performed using the BioEstat 5.0 program. For the confirmation of the *Tm* for each NTM strain, we used the ANOVA variance with the Tukey test.

Sensitivity and specificity values were calculated to evaluate the real-time multiplex PCR-HRM system. The kappa index was used to evaluate the degree of agreement between the tests. Calculations were performed using the OpenEpi version 3.1 statistical software.

### Ethics approval

The study was approved by the ethical committee of the Aggeu Magalhães Institute, Oswaldo Cruz Foundation CAAE: 07382012.4.0000.5190.

## RESULTS

A total of 79 specimens were tested for the presence of mycobacteria during the study period. Multiplex real-time PCR with high-resolution melting (HRM) results as well as the results of DNA sequencing, which was used as the gold standard method assay, are shown in Table 2. NTM species were identified in 70 (88.60%) of the samples and the M. tuberculosis complex was identified in 9 (11.40%) of the samples.

**TABLE 2:** Comparison between real-time PCR-HRM and sequencing of specific genes of the studied Mycobacterial species.

Species identified by real-time multiplex PCR-HRM	Species identified by specific gene sequencing								
	*M. avium*	*M.intracellulare*	*M. kansasii*	*M.abcessus*	*M. fortuitum*	*M. tuberculosis*	NTM	Not identified	Total
*M. avium*	4								
*M. intracellulare*									
*M. kansasii*			35						
*M. abcessus*				8					
*M. fortuitum*					6				
*M. tuberculosis*	1		2	2	1	9	1		
NTM							4		
Not identified	2				4				
Total	7	0	37	10	11	9	5	0	79

**NTM:** Nontuberculous mycobacteria.

The *Tm* averages obtained for each species in the real-time multiplex PCR-HRM are presented in Table 1; due to technical problems, it was not possible to standardize the *Mycobacterium chelonae*. There was a significant statistical difference when comparing the *Tm* values of all species used in the study (p <0.01).

**FIGURE 2:**
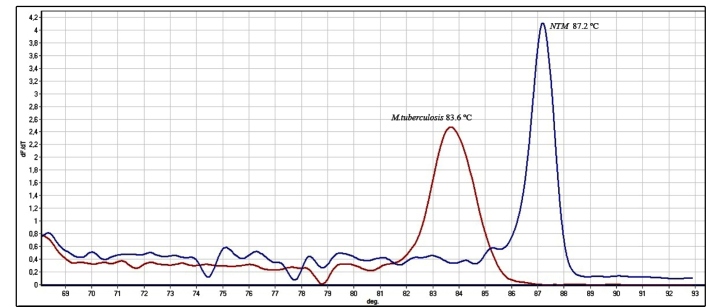
Analysis of the curve of real-time multiplex PCR-HRM: *M. tuberculosis* and NTM.

 Step 1 of the multiplex real-time PCR-HRM assay was performed on the 79 (100%) mycobacterial culture isolates. It detected 63 (79.74%) NTM and 16 (20.26%) *M. tuberculosis* complex species*.* The sensitivity and specificity were 90% (CI = 80.77-95.07) and 100% (CI = 70.8-100), respectively, with an accuracy value of 91%. Specific gene sequencing and real-time multiplex PCR-HRM showed good agreement, as demonstrated by a kappa index of 0.67 (p <0.0001) (Figure 2).

The 63 clinical isolates identified as nontuberculous mycobacteria in Step 1, proceeded to Steps 2 and 3 of the real-time multiplex PCR-HRM for the differentiation of NTM species. In step 2, 35 (55.55%) isolates were identified as *M. kansasii*, 8 (12.69%) as *M. abscessus*, and 4 (6.37%) as *M. avium*. The sensitivities were 94.59% (CI = 82.3-98.5), 80% (CI = 49.02-94.33) and 57.14% (CI= 25.05-84.18), respectively (Table 2). The specific gene sequencing and multiplex real-time PCR-HRM showed good agreement, as demonstrated in each species identified by the kappa index: k 0.935 (p < 0.001) for *M. kansasii*, k 0.871 (p < 0.001) for *M. abscessus*, and k 0.571 (p < 0.001) for *M. avium*.

In Step 3, only *M. fortuitum* species were identified in 6 (9.52%) isolates, showing a sensitivity of 54% (CI = 25.05-84.18) and a kappa index of 0.665 (p < 0.001). It was not possible to identify 10 mycobacterial isolates (15.87%) at the end of all stages of the multiplex real-time PCR-HRM assay. The sequencing identified 4 isolates as *M. fortuitum*, 2 as *M. avium*, 1 as *M. wolinsky*, and 3 as *M. celeriflavum.* The multiplex real-time PCR-HRM assay did not have specific primers for the identification of *M. wolinsky* and *M. celeriflavum* (Table 2).

## DISCUSSION

In the present study, we aimed to modify the combination of the primers used in the three different steps of real-time multiplex PCR-HRM, previously published^13^_._ Initially, we differentiated between *M. tuberculosis* and NTM, at stage 1 of PCR, because this region presents high tuberculosis endemicity, since the northeastern region is the second in Brazil in new cases of tuberculosis^21^ ^,^ ^22^, reducing the number of species identification necessary in the next steps (2 and 3). In Steps 2 and 3, accurate identification was performed for NTM species with the highest prevalence in the region (*M. avium, M. intracellulare, M. kansasii, M. abcessus, and M. fortuitum*). An advantage of this system is the PCR conditions of the reaction: Steps 1, 2, and 3 were designed to be equivalent and the reactions could be performed simultaneously while the assay was run. The assay was able to identify approximately 84% of the NTM species evaluated, with precise identification of species and subspecies evaluated.

The multiplex real-time PCR-HRM was able to perform species differentiation using different melting temperatures. In Step 1, it was observed that of the 16 species identified as *Mycobacterium tuberculosis* complex by multiplex real-time PCR-HRM, 7 were NTM species isolates, found by specific gene sequencing (2 *M. kansasii,* 2 *M. abcessus,* 1 *M. fortuitum,* 1 *M. avium*, and 1 *M. interjectum*), which were characterized as false positives. Kim and collaborators^23^ explain that it can be difficult to interpret melting curves in cases involving mixed sequences or strains with a variation in the target sequences. They found that the reactions of the hydrolysis probe assay have advantages over the assay that employs melting curve analysis because the analyses of amplification curves are simpler.

Of the 37 *M. kansasii* isolates identified by specific gene sequencing, the multiplex real-time PCR-HRM, Step 2, identified 35 as *M. kansasii* and 2 as *M. tuberculosis* (considered false-positive), in which solid culture identified these 2 isolates as slow-growing. Of the *M. avium* isolates, 7 species were identified by sequencing. However, the multiplex real-time PCR-HRM identified 4 isolates as *M. avium* 1 as *M. tuberculosis* (considered false positive), which was identified by solid culture as slow-growing, and 2 were not identified in any of the steps of PCR. The *16S rRNA* gene was the molecular target used for the design of primers to detect NTM species*.* However, this gene has highly similar sequences in different *Mycobacterium* species, so it may not distinguish closely related species. To overcome these limitations, efforts have focused on the use of other genes such as *hsp65* and *rpoβ* for accurate identification of NTM species^24^. Sampling variability associated with low concentrations of 251 and 16S target sequences, both of which occur as single copies in the genomes as well as the differences in amplification efficiency, may explain the differences in detection^25^.

In the *M. abcessus* isolates, 10 were identified by sequencing, but the multiplex real-time PCR-HRM identified 8 as *M. abcessus* and 2 as *M. tuberculosis* (considered false positive). Specific gene sequencing identified 11 species of *M. fortuitum*, in which 6 of these were identified in multiplex real-time PCR-HRM, 1 was identified as *M. tuberculosis* (false-positive result), and 4 were not identified in any of the steps. However, the 2 isolates of *M. abcessus* and *M. fortuitum* were identified in the solid culture as rapid-growth. The discordant results may be due to the genetic variability in the molecular target, the limited specificity of primers in the clusters used, or the limitation of the efficient PCR methodology used, highlighting the importance of further studies with different cluster profiles^24^.

It was not possible to identify 4 mycobacterial isolates by multiplex real-time PCR-HRM, because there are no specific primers for these species (*M. wolinski* and *M. celeriflarum)*. This isolate was identified only by specific gene sequencing (1 as *M. wolinski* and 3 as *M. celeriflarum*), considering the non-prediction of mycobacterial isolation of these species in the studied region. Although the multiplex real-time PCR-HRM assay was able to identify the *M. intracellulare, M. chelonae*, and *M. smegmatis* species, sampling did not confirm these species. The kappa index assessed at all stages of mycobacterial identification maintained the classification between strong (substantial) and moderate.

Studies have used real-time PCR techniques to differentiate between NTM infections and TB^17^ ^,^ ^20^ ^,^ ^23^. Chen et al.^24^ demonstrated that an in-house PCR-HRM assay targeting two genes, *16S rRNA* and *hsp65*, could successfully differentiate at least 14 NTM species, including 8 slow- and 6-rapid growth, of clinically relevance.

The molecular differentiation of NTM species has led to recent advances, mainly due to the recognition of the importance of these bacteria with pathogenic potential to humans. Studies of methods and molecular markers, which discriminate between NTM species, are scarce in the scientific literature. Therefore, research is urgently required. However, we concluded that the use of a variety of genetic targets applied in molecular diagnostic testing provides greater precision in differentiating between the *Mycobacterium tuberculosis* complex and NTM. Thus, the identification of NTM species through routine molecular diagnostic tools is an alternative to the rapid diagnostic definition of this infection. Even though cumbersome and expensive to use routinely, it is necessary due to the burden on public health.

In this study, it was possible to differentiate between TB and NTM infections with precision, demonstrating its usefulness as a tool for the rapid differential diagnosis of TB and NTM disease. In order to characterize the NTM species, despite the limited sensitivity, an alternative and cheaper technique than the sequencing standard can be considered, mainly for the identification of the species *M. abscessus, M. fortuitum,* M. avium complex*, M. kansasii.*

The multiplex real-time PCR-HRM modified assay has the potential to identify rapid and efficient nontuberculous mycobacteria of clinical importance, which is crucial for immediate implementation of the appropriate therapy and thus avoiding complications and sequelae in patients.
